# Sleep in Different Contexts: The Perspectives of European Psychology

**DOI:** 10.1192/j.eurpsy.2025.81

**Published:** 2025-08-26

**Authors:** J. F. Leader

**Affiliations:** European Federation of Psychologists’ Assocations (EFPA), Brussels, Belgium

## Abstract

**Abstract:**

Sleep is not something that occurs in isolation—it is embedded in a context. These contexts include biological, psychological and social factors, as well as a range of environmental considerations.

Throughout Europe psychologists engage in research and practice and collaborate with colleagues from allied professions such as psychiatry and those with lived experience on the topic of sleep.

Insights are gathered from throughout the network of the European Federation of Psychologists’ Associations (EFPA). Individual examples and emergent themes are identified from a wide range of contexts including:

The role of sleep in European policy

Sleep in times of crisis

Community-oriented approaches to supporting healthy sleep patterns

The role of sleep and rest in the workplace

How concern over topics like climate change affects sleep

Competency around sleep in practitioner training and standards

The implications of digitalisation on sleep

**Disclosure of Interest:**

None Declared

